# Blue-Black or White-Gold? Early Stage Processing and the Color of 'The Dress'

**DOI:** 10.1371/journal.pone.0161090

**Published:** 2016-08-31

**Authors:** Jeff Rabin, Brook Houser, Carolyn Talbert, Ruh Patel

**Affiliations:** Rosenberg School of Optometry, University of the Incarnate Word, San Antonio, Texas, United States of America; University of Glasgow, UNITED KINGDOM

## Abstract

**Purpose:**

In Feb 2015 an image of a dress posted on Tumblr triggered an internet phenomenon: Is the Dress blue and black (BB) or white and gold (WG)? Many claim BB and others insist WG while the true colors are BB. The prevailing theory is that assumptions about the illuminant govern perception of the Dress with WG due to bluish lighting and BB due to yellowish. Our purpose was to determine if early stage optical, retinal and/or neural factors also impact perception of the Dress.

**Methods:**

Thirty-nine subjects were categorized as BB or WG based on their initial perception of the Dress and their perception reported when viewing the Dress on iPhone 5, iPad, and 22” LCD displays. Macular pigment optical density (MPOD) measured with the QuantifEye™ MPS II and visual brainwaves (VEPs) in response to brief presentations of a transparency of the Dress illuminated by a flashing light were measured on each subject and compared between BB and WG groups. Additionally, CIE chromaticity (color) and luminance (brightness) were measured from multiple areas of the Dress image to determine cone stimulation and contrast.

**Results:**

Mean MPOD was higher in the WG group (0.49) vs. the BB (0.41, p = 0.04) and median values were higher as well (WG = 0.46, BB = 0.36, p = 0.03). There was no difference in VEP amplitude between groups (p > 0.85) but mean VEP latency was longer in WG (130 msec.) vs. the BB group (107 msec., p = 0.0005). Colorimetry of the Dress showed significantly greater stimulation of blue cones (contrast = 73%) vs. red and green sensitive cones (contrast = 13%).

**Conclusions:**

Our findings indicate that observers with denser MPOD may be predisposed to perceive the Dress as WG due to great absorption of blue light by the macular pigment. Moreover, the novel, substantial stimulation of blue cones by the Dress may contribute to ambiguity and dichotomous perception since the blue cones are so sparse in the retina. Finally, the delayed WG VEPs indicate distinct neural processing in perception of the consistent with fMRI evidence that the WG percept is processed at higher cortical levels than the BB. These results do not fully explain the dichotomous perception of the Dress but do exemplify the need to consider early stage processing when elucidating ambiguous percepts and figures.

## Introduction

**Fig 1:**
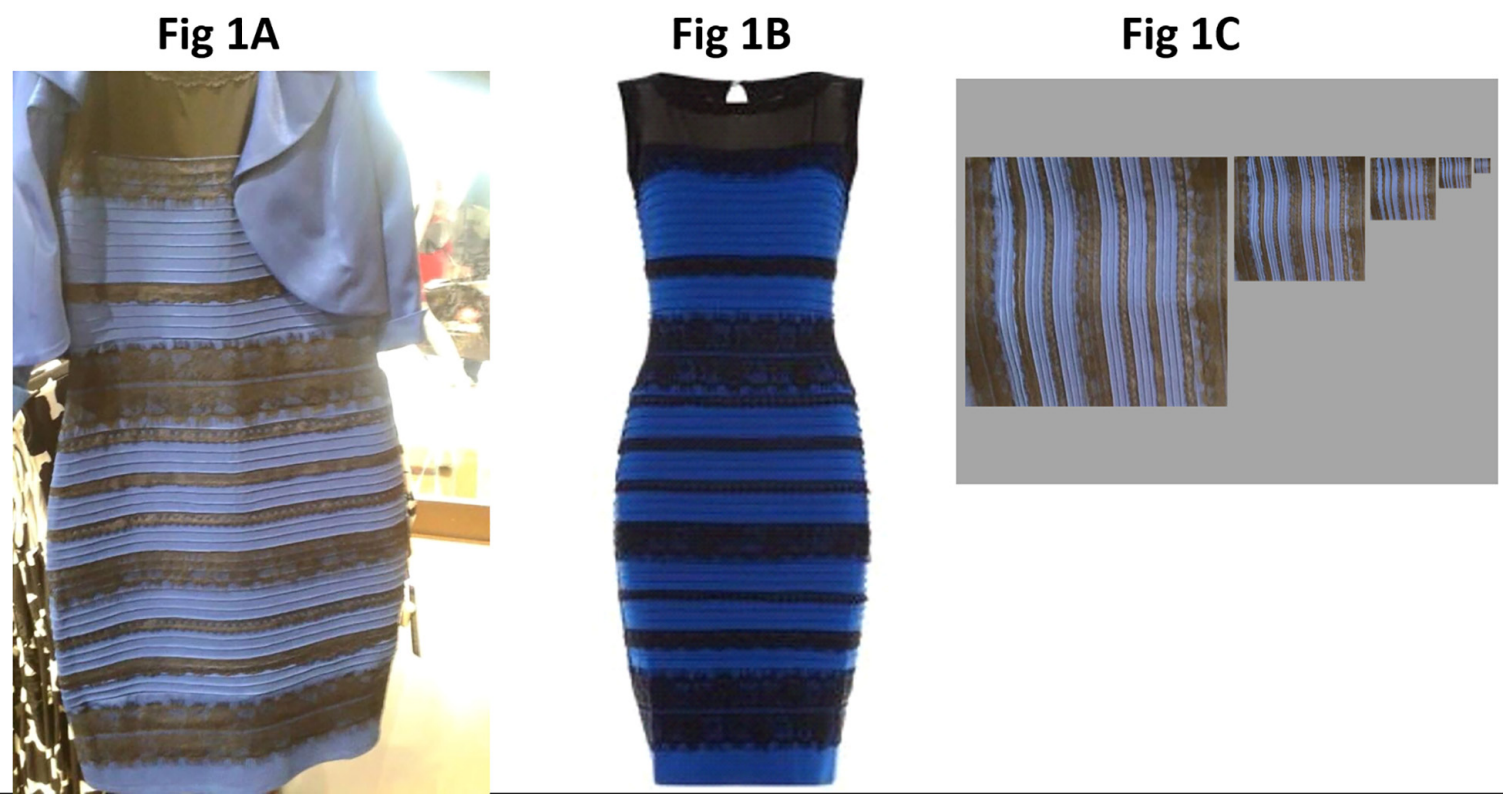
The Dress as seen on the internet shown in A and the actual blue and black dress is shown in B. C shows an extracted image of the Dress consisting of vertical stripes of decreasing spatial frequency that was used in the present study to explore perception of the dress with limited contextual cues.

In February 2015 an image of a dress was posted on Tumblr (Fig 1A) which triggered an internet phenomenon debating the issue: Is the Dress blue and black (BB) or white and gold (WG)? Many claim the Dress is BB while others are convinced it is WG. Fewer perceive The Dress to be intermediate (e.g., light blue and brown or burnt gold) while the actual colors of the Dress are blue and black (Fig 1B). This unprecedented dichotomy in color perception prompted worldwide opinions from scientists, politicians and celebrities alike. Initial responses from vision scientists and a current prevailing view is that the percept depends, at least in part, on one’s inherent assumptions about how the dress is being illuminated, predicated on our knowledge of color constancy. It is well established that perceived hues remain relatively constant regardless of changes in the illuminant[1–3]. Hence if one assumes that, in the Dress image, the illuminant is bluish in coloration (e.g., midday indirect sunlight, fluorescent lighting), then the perception is likely to be WG because the visual system discounts the shorter wavelength, bluish contribution resulting in the perception of a white and gold dress illuminated by bluish light. Conversely, if one assumes that the illuminant is more yellowish in color (e.g., indirect sunlight at day’s end, incandescent lighting), then the perception tends to be BB because the visual system discounts the longer wavelength contribution to the darker stripes on the dress and interprets the lighter stripes as blue and darker stripes as black. This explanation has received considerable support from experts in our field and certainly must play a role in the BB vs. WG perception[4–7]. Additional contributing factors include the tendency to perceive light or pastel blues as grey or white[7], which further explains the dichotomous perception, as well as more recent studies indicating separate cortical processing for WG vs. BB[8] and effects of pupil size and possibly retinal illuminance on the percept[9]. However, why some individuals perceive the Dress to be BB and others perceive WG remains enigmatic. Are there factors which predispose individuals to perceive BB vs. WG? Our purpose was to determine whether visual system “front-end” factors, including macular pigment optical density (MPOD), relative stimulation of L, M and S cones, and early stage cortical processing, help explain the dichotomous perception of the Dress.

## Methods

Subjects were recruited from the students, faculty, and staff at the University of the Incarnate Word Rosenberg School of Optometry. All subjects had visual acuity of at least 20/20 and no history of ocular disease. The study protocol was approved by the University of the Incarnate Word Institutional Review Board and all subjects were briefed on the protocol and provided written informed consent prior to participation in the study in accord with the Declaration of Helsinki. Subjects included 39 visually normal observers (mean age ± SD = 32 ± 10 years, 20 males). Initially each subject was asked whether she/he had seen the image of the Dress on the internet and what their initial perception was: BB, WG, or other such as light blue and brown/burnt gold. Five subjects had not seen the image previously: three reported BB, one WG and one blue/burnt gold after viewing our displays. A total of 19 subjects perceived BB and 17 WG based on their initial perception. Three reported light blue/brown-burnt gold; these three subjects were not included in the main analysis and their data were intermediate between groups.

In addition to their initial perception, we asked each subject to report on the colors of the Dress while viewing the internet image (Fig 1A) on a 22” computer display, iPad tablet and iPhone 5S display and an extracted version of the Dress portrayed as vertical stripes of increasing spatial frequency lacking contextual cues from the internet image (Fig 1C). Each display was set at default settings and viewed in a dimly lit room at approximately 60 cm with order of presentation counter-balanced across subjects.

Macular pigment, which selectively absorbs short wavelength light, is concentrated in the central retina, varies between individuals, and is modifiable by diet[10,11], was measured in right and left eyes of each subject using the computer-controlled QuantifEYE™ MPS II compact desktop system described in detail in prior studies[12,13]. The MPS II uses a modified heterochromatic flicker photometric technique to determine the green/blue (530/465 nm) luminance ratio to achieve minimum perception of flicker for a 1 degree foveally fixated target compared to a 2 degree extra-foveal target 8 degrees from fixation. Since the green light is absorbed negligibly by macular pigment, the log difference between blue light absorption to achieve minimum flicker in the fovea compared to the extra-foveal site quantifies macular pigment optical density (MPOD). Subjects were optimally corrected during testing and all measurements were deemed valid (“acceptable”) by the MPS II system.

In addition to MPOD, onset VEPs were recorded binocularly from each subject in response to onset presentations of the dress. Prior to testing, each subject’s scalp and earlobes were cleaned with alcohol and abrasive cleaner and the VEP gold cup active electrode was filled with conductive paste and taped 1 cm above the inion with reference and ground ear-clip electrodes filled with conductive paste and affixed to each earlobe. Each subject wore an elastic headband to secure the active electrode in place and electrode impedance was maintained at ≤5 kilohms. The VEP stimulus was a high resolution transparency of the original dress image retro-illuminated by a flashing neutral white background (100 cd/m^2^) from a calibrated VEP monitor (Diagnosys, LLC). The dress stimulus subtended an angle of 12.2° x 16.2° degrees and was viewed binocularly at 1m in a darkened room with subjects optimally corrected for the viewing distance. The dress appeared two times per second, with each presentation lasting 250 msec. followed by a 250 msec. black field. The VEP was recorded for 200 msec. at the onset of each dress presentation. Each signal was amplified 8X, band-pass filtered (1–30 Hz), and the system computed the average VEP to 70 pattern (dress) onsets. Prior to testing, each subject adapted to the white background for about 6 minutes during electrode application. The average VEP waveform in response to 70 pattern (dress) onsets was recorded twice from each subject and amplitude in microvolts (μV) vs. latency (msec.) were exported as digital values to compare VEPs across BB and WG groups.

To quantify colorimetric values of the Dress, the internet image was displayed on the 22” monitor and components of the dress which appeared to be in shadow, fully illuminated by ambient light, and moderately illuminated were magnified and measured with a Spyder 4 colorimeter (Datacolor, Lawrenceville, NJ). This system, which is designed for calibration of electronic displays, was equipped with a custom program to transform display luminance and CIE chromaticities to cone excitations based on Smith and Pokorny[14] cone fundamental sensitivities and equations specified by Wyszecki and Stiles[15] and Cole and Hine[16] and utilized in the development of color vision tests[17]:

$$L_{excitation}=Luminance\left[\frac{0.15514x}{y}+0.54312-\frac{0.03286(1-x-y)}{y}\right]$$

$$M_{excitation}=Luminance\left[\frac{0.15514x}{y}+0.45684+\frac{0.03286(1-x-y)}{y}\right]$$

$$S_{excitation}=Luminance[0.00801(1-x-y)/y]$$

**Fig 2:**
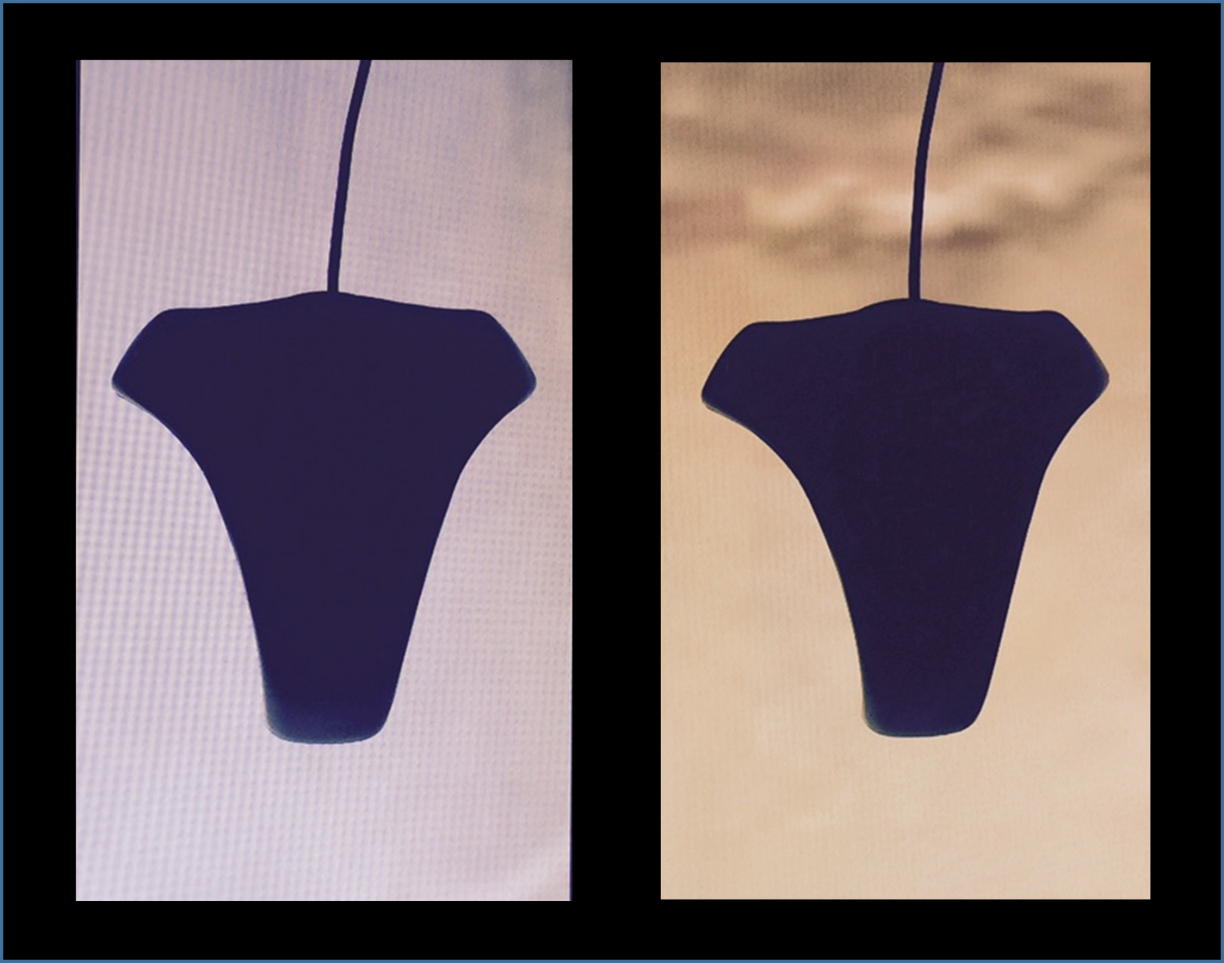
The colorimeter positioned over the 22” LCD display with magnified components of the Dress image. The system measures luminance and CIE chromaticity.

Cone excitations were used to compute cone contrasts and additional metrics to determine the relative contributions of L, M and S cones as well as opponent mechanisms. Fig 2 shows the colorimetric set-up to quantify luminance and chromaticity.

## Results

The vast majority of subjects reported no difference in their BB vs. WG perceptions between the iPhone, iPad, 22” LCD display, and extracted stripe images of the Dress (Fig 1C). One BB subject reported that the tablet Dress appeared blue and gold, another BB reported that the stripe pattern appeared blue and gold, and one WG subject reported that the stripes appeared blue and gold. Hence the majority of observers perceived the same Dress colors regardless of display.

**Fig 3:**
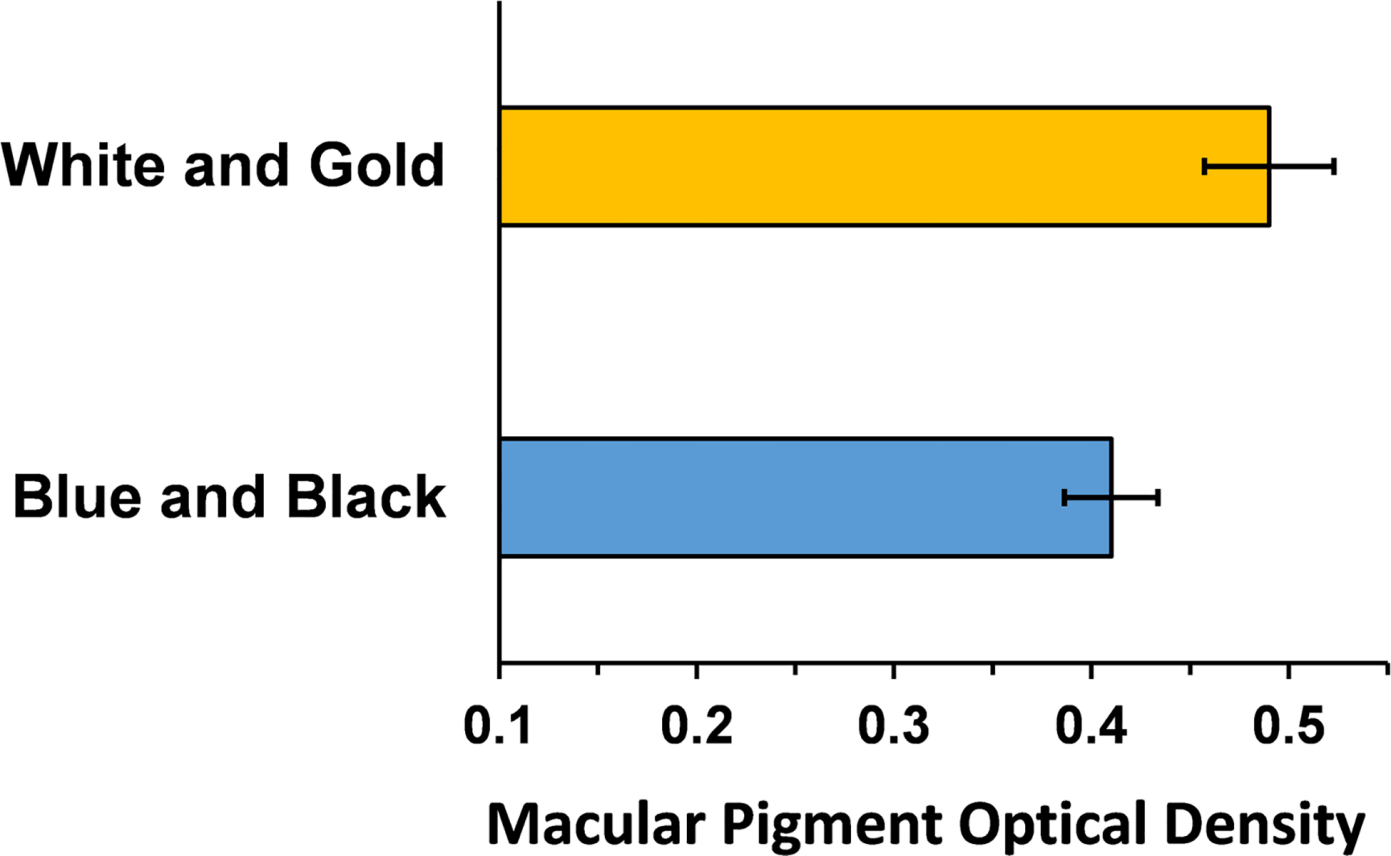
Mean (±1 SE) MPOD is plotted for subjects who perceived the Dress to be white and gold (n = 17) or blue and black (n = 19). The difference between means is significant (p < 0.04).

MPOD was not significantly different between subjects’ right and left eyes (p > 0.38); hence the mean value of right and left eyes for each subject was used for analysis. Fig 3 shows that the mean MPOD was significantly higher in the WG group (mean = 0.49) compared to the BB group (mean = 0.41; two-tailed t-test, t = 2.14, p = 0.0395). Non-parametric comparison of the median values showed a comparable result. The median MPOD for the WG group (0.46) was higher than the median MPOD for the BB group (0.36) and this difference was significant (2-tailed Mann-Whitney U, p = 0.0295)[18]. These findings suggest that greater pre-retinal absorption of short wavelength light by the macular pigment may predispose some observers to perceive the Dress as WG rather than BB.

**Fig 4:**
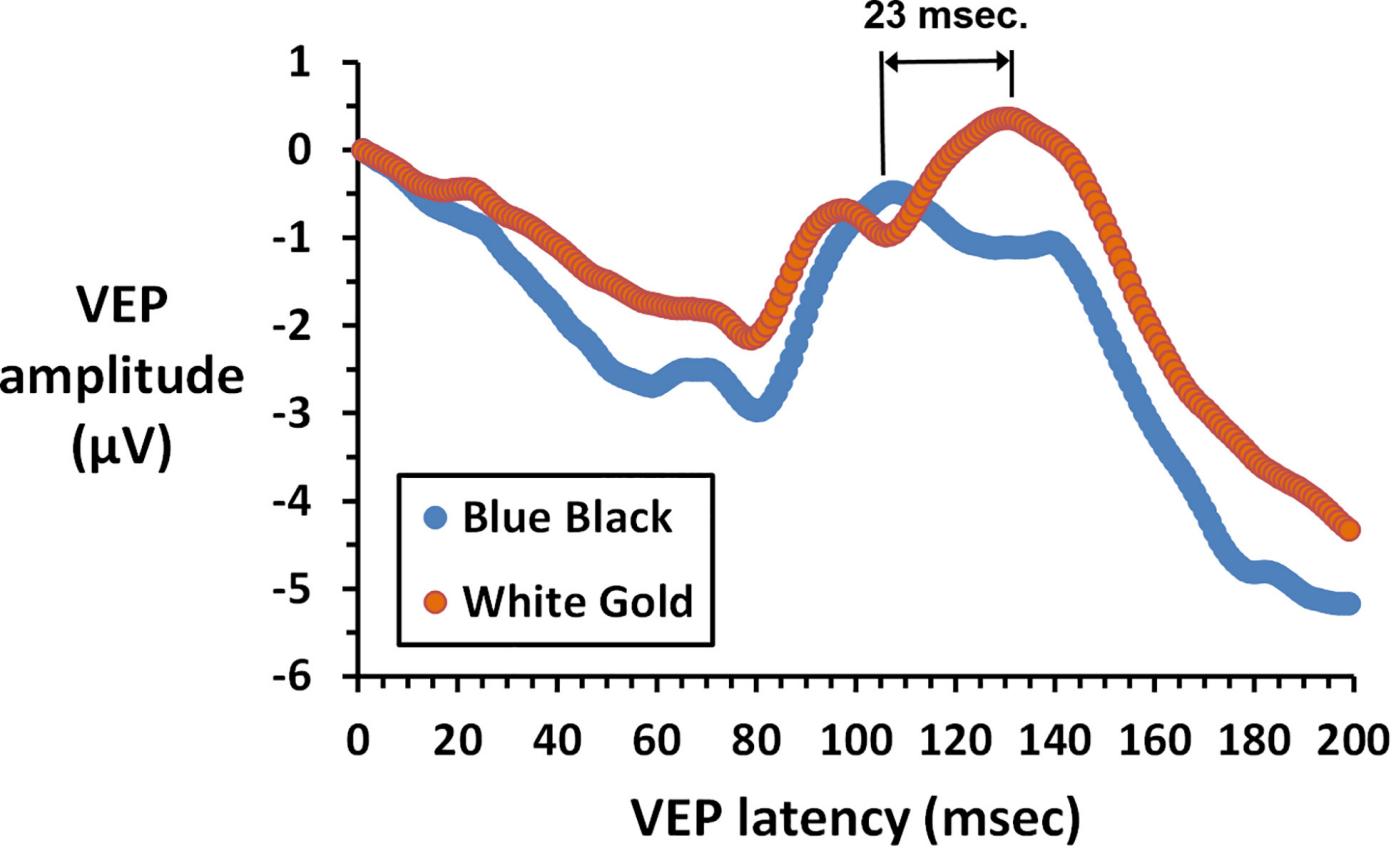
The VEP was digitized for each subject and averaged across all WG (n = 17) and BB subjects (n = 19). The latency to the positive peak was significantly longer in the WG group (p = 0.0005).

The VEP data from each subject were exported as a digital file with amplitude (μV) at each msec. of recording. The amplitudes at each msec. were then averaged across BB and WG subjects to derive a mean VEP waveform for each group. Fig 4 shows mean VEPs for BB and WG groups. As illustrated, onset VEPs to the appearance of the Dress showed a negative wave followed by a positive peak. There was with no significant difference between VEP amplitudes for the WG group (mean = 6.6 μV) vs. the BB group (mean = 6.4 μV) when measured as the most negative trough to the subsequent most positive peak (two-tailed t-test, t = 0.189, p > 0.850). However, there was a significant difference in latency to the positive peak with mean latency for the WG group (130 msec.) significantly longer than mean latency for the BB group (107 msec., two-tailed t-test, t = 3.845, p = 0.0005, Fig 4). As in the case of MPOD, nonparametric comparison of median latencies also revealed a significant increase in the WG group (median latency = 132 msec.) vs. the BB group (median latency = 105 msec., p = 0.0008). These findings demonstrate unique VEP latencies for WG and BB suggesting differences in processing time for WG vs. BB perceptions. However, regression analysis revealed no significant correlation between MPOD and VEP latency at this time (F = 0.238, p>0.628).

Colorimetric evaluation of various components of the Dress on the 22” computer display used for the perceptual analysis showed considerable variation in the luminance of the Dress components depending on whether the image appeared brightly illuminated or in shadow with blue (or white) components varying from 51 to 20 cd/m^2^ while the black (or gold) components varied from 25 to 8 cd/m.^2^ Table 1 shows luminance, CIE chromaticity, and L, M and S cone excitations for blue and black components of the middle portion of the Dress based on measurements (Fig 2) and equations described earlier for computation of cone excitations. Contrast values are based on Derrington-Krauskopf-Lennie (DKL) color space which assumes three orthogonal cardinal axes: (a) L-M isoluminant axis along which L and M cone stimulation varies in reciprocal fashion such that luminance (L+M) and S cone stimulation remain constant, (b) S cone isoluminant axis along S cone stimulation varies from high to low while L and M cone stimulation and hence luminance (L+M) remain constant, (c) an orthogonal achromatic axis along which L, M and S cones are stimulated equally[19,20]. The bottom three rows of Table 1 show Michelson contrast values for L-M, S cone, and luminance mechanisms:

$$L-M:\;100[{\left(L-M\right)}_{blue}-{\left(L-M\right)}_{black}]/{\left[(L-M\right)}_{blue}+{\left(L-M\right)}_{black}]$$

$$S_{cone}:\;100[{(S}_{blue}-S_{black})]/{(S}_{blue}+S_{black})]$$

$$Luminance:\;100[{\left(L+M\right)}_{blue}-{\left(L+M\right)}_{black}]/{\left[(L+M\right)}_{blue}+{\left(L+M\right)}_{black}]$$

**Table 1:** Luminance and Colorimetric Evaluation of ‘The Dress.’

Measurement	Dress Component	
	Blue	Black
Luminance (cd/m^2^)	50.85	31.48
CIE chromaticity (*x*,*y*)	*0*.*27*, *0*.*28*	*0*.*40*, *0*.*42*
L cone excitation	32.68	21.32
M cone excitation	18.16	10.16
S cone excitation	0.65	0.10
L—M	14.52	11.16
L—M contrast	13.08%	
S cone contrast	73.33%	
Luminance contrast	23.53%	

Note that in luminance equation values are in fact equal to L+M values for blue and black. While all three canonical post-receptoral mechanisms are likely to contribute to perception of the Dress, the high stimulation of the S cone (koniocellular) pathway may constitute a source of ambiguity which conceivably may contribute to the dichotomous perception of the Dress.

**Fig 5:**
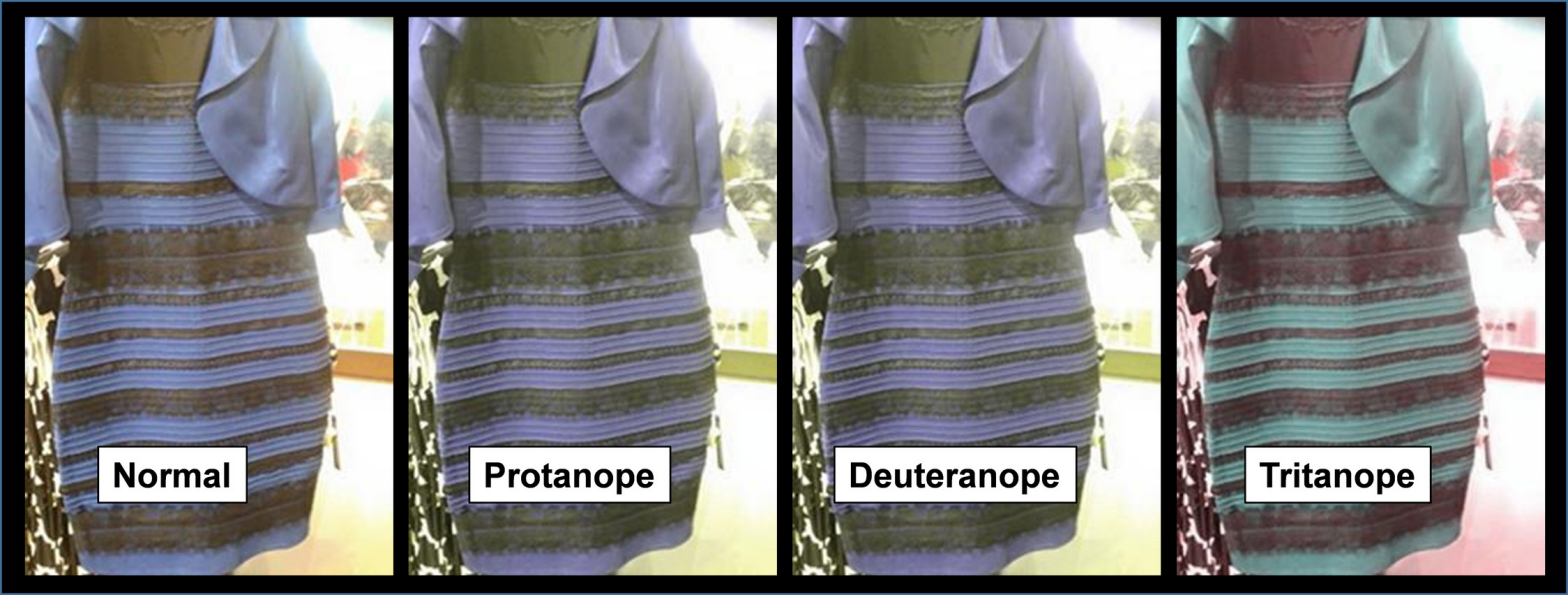
The Dress as seen by a color vision normal observer, protanope, deuteranope and tritanope. Lacking S cones has the greatest impact on dress color. See text for further details.

The importance of S cone input in perception of the dress is further illustrated in Fig 5 which illustrates renderings of the Dress as seen by a protanope, deuteranope and tritanope (hereditary absence of L, M or S cones, respectively; www.color-blindness.com/coblis-color-blindness-simulator). Lacking L or M cones has minimal impact on perceived dress colors while a lack of S cones yields a very different perception suggesting a primary role of the S cone input in perception of the Dress.

## Discussion

Our results indicate that early-stage optical, retinal and neural factors influence perception of the Dress. Observers with denser macular pigment tend to see the Dress as WG while those with less dense pigment see BB. This suggests that greater pre-retinal absorption of short-wavelength light may predispose observers to see WG vs. BB. Additionally, colorimetric analyses, coupled with renderings of the dress without input from L or M cones, indicate a strong role of S cone input in perception of the Dress. It is conceivable that this rare combination of strong stimulation S cones vs. weaker stimulation of L and M cones, as well as the post-receptoral L-M and luminance pathways, contributes uncertainty making it more difficult for observers to disambiguate the Dress from the illuminant. However, it seems unlikely that the strong S cone component determines the actual colors perceived. Finally, VEPs in response to onset presentation of the Dress showed comparable waveforms for BB and WG, but a prolonged latency to the positive peak for WG observers. This provides an objective, neural index of the difference in perception and is in general agreement with a recent fMRI study which showed that in WG observers the Dress image produces higher activation in cerebral areas mediating higher cognition, including frontal and parietal brain cortex[8].

Limitations of this study include the relatively small number of subjects tested. A larger sample size may provide more definitive evidence for macular pigment and VEP differences. While our results add to the growing body of knowledge regarding perception of the Dress and offer factors which may predispose observers to see WG vs. BB, we offer no definitive basis for the dichotomy in perception.

As noted earlier, a prevailing theory to explain the dichotomous perception of the Dress involves assumptions about the illuminant, wherein observers who assume the illuminant is broad-band but weighted toward shorter wavelengths perceive WG, interpreting the bluish hue to the illuminant reflecting from a white or neutral fabric and perceiving the goldish component veridically. Conversely, those who assume a broad-band illuminant weighted toward longer wavelengths perceive BB, interpreting the lighter stripes as blue and darker stripes as black by discounting the longer wavelength contribution to the darker stripes on the Dress[4–7]. Winkler and colleagues[7] enhanced this interpretation by showing that pastel blues are often perceived as grey or white while yellows are perceived veridically to further explain the dichotomy. As noted earlier, Schlaffke and colleagues[8] localized WG perception to anterior cortical areas involved in cognition and argue that top-down perception plays a role in the perceptual dichotomy, while Vemuri and colleagues[9] found that WG perception of the dress is associated with smaller pupil sizes, an additional “front-end” factor comparable to what we report herein.

Inherent assumptions about the illuminant and cognition undoubtedly play a significant role in dichotomous perception of the Dress, but why some see BB and others WG remains unclear. The findings reported herein, suggesting that MPOD and cone input may influence perception of the Dress, emphasize the need to consider “front-end” factors when elucidating ambiguous visual perceptions, including those which often occur in acquired and senescent brain disease. It is hoped that our findings will better elucidate these perceptions and associated behaviors paving the way for improvements in treatment.

## Supporting Information

S1 File Macular pigment optical density and VEP mean data. (XLSX)
